# When Is It Helpful to Get Involved? Adolescents’ Perceptions of Constructive and Aggressive Bystander Support from Friends, Acquaintances, and Public Figures in Cyberbullying

**DOI:** 10.3390/ijerph21091142

**Published:** 2024-08-28

**Authors:** Karissa Leduc, Megha Pooja Nagar, Oksana Caivano, Victoria Talwar

**Affiliations:** Department of Educational and Counselling Psychology, McGill University, Education Building, 3700 Mc Tavish St, Montreal, QC H3A 1Y2, Canadavictoria.talwar@mcgill.ca (V.T.)

**Keywords:** cyberbullying, bystanders, support, relationships, adolescents

## Abstract

The present study examines adolescents’ perceptions of both constructive and aggressive forms of bystander support and how these perceptions differ according to whether an acquaintance of the target, a friend of the target or a public figure is providing it. Ninety-nine adolescents between 13 and 17 years old (*M*_age_ = 14.42; *SD* = 1.35) participated in this study. Adolescents viewed a total of nine videos, each depicting a public cyberbullying situation on Instagram and a form of constructive or aggressive bystander support from an acquaintance, a friend, or a public figure in relation to the target of cyberbullying. After each video, adolescents were asked how helpful or hurtful the bystander’s form of support was on a Likert-type scale. A significant relationship was found between the bystander’s relationship to the target, the form of support and the helpfulness of bystander support. Overall, support from friends was perceived as helpful regardless of whether it was constructive or aggressive. Moreover, it was seen as harmful for acquaintances to engage in aggressive behaviours in support of targets of cyberbullying, but generally helpful for public figures to engage in those same behaviours.

## 1. Introduction

Digital media plays an important role in adolescents’ lives. Ninety-two percent of teenagers go online almost every day, and approximately 71% of teenagers report having profiles on multiple social media platforms such as Facebook, Snapchat, and Instagram [1]. As adolescents spend more time on social media, they are increasing their online social interactions but also their risk of exposure to cyberbullying [2]. Exposure to cyberbullying has been linked to decreased well-being by reducing self-esteem, increasing depression and social isolation, and diminishing self-efficacy [3]. Fortunately, bystanders have taken centre stage in recent years as agents of change in cyberbullying because they can engage in different forms of intervention, either directed towards supporting the target (constructive interventions) or directed towards attacking the cyberbully (aggressive interventions) [4]. Moreover, due to the nature of public social media activity on platforms such as TikTok or Instagram, bystander intervention can come from public figures (e.g., influencers, celebrities, etc.) in addition to acquaintances or friends of a target [5,6]. While research has shed light on different forms of bystander support and their determinants, little is known about how adolescents perceive the helpfulness of those forms of support and whether their perceptions vary depending on the parties involved. Accordingly, based on Ouvrein et al.’s (2019) [5] cyberbullying experience and behavioural coping frames, the current study used hypothetical cyberbullying scenarios to examine adolescents’ perceptions of the helpfulness of a bystander’s different constructive and aggressive behaviours in support of targets of cyberbullying. This study also examined how adolescents’ perceptions differed according to the bystander’s relationship with the target of cyberbullying (i.e., whether they were a friend, an acquaintance, or a public figure).

### 1.1. Support from Bystanders in Cyberbullying

Bystanders are a constant presence on social media. Their responses to cyberbullying have been distinguished as positive and constructive or negative and aggressive in previous research (e.g., [4,7]). Bystanders can be known to engage in constructive support by engaging in positive behaviours that promote both the well-being and sense of safety of users on social media and of targets of cyberbullying [4,8]. For instance, bystanders can provide constructive support to targets by asking the cyberbully to stop or by providing emotional support to the target following the event [5]. However, bystanders can also engage in aggressive behaviours that can contribute to feelings of discomfort among users on social media and targets of cyberbullying [4,9]. For instance, they can provide aggressive support to the target by “biting back” at the cyberbully or exposing their behaviour publicly [5]. While these behaviours may be performed with the intention to protect a target of cyberbullying, they can be harmful towards the perpetrator of cyberbullying and contribute to the cycle of cyberbullying online.

While prevention activities generally target intervention from bystanders of cyberbullying [10], it is important to understand how adolescents perceive different forms of bystander support online for two main reasons: (1) to understand if the interventions promoting support from bystanders are actually perceived as helpful by adolescents who are likely to be targets of cyberbullying and (2) to know if they are likely to engage in similar behaviours to support targets of cyberbullying as suggested by the Bystander Intervention Model [11].

According to the Bystander Intervention Model [11], a bystander’s response to cyberbullying is influenced by how they evaluate the event in question. For instance, if an adolescent notices an act of cyberbullying that they do not perceive as harmful, they may be less likely to provide support to the target. On the other hand, if they perceive the online interaction to be harmful, they may be more likely to ask the cyberbully to stop or to provide emotional support. Latané & Darley (1970) [11] also suggest that the perceived helpfulness of a chosen intervention in support of a target of aggression is influential in a bystander’s decision to intervene. While the Bystander Intervention Model was initially developed to understand bystander behaviour in face–to–face emergency situations, it has found empirical support in online mediated contexts (e.g., [12]. Moreover, similar to the Bystander Intervention Model, the social cognitive theory of moral action also suggests that how an individual perceives a situation will influence their responses to it [13,14]. Thus, it is important to examine adolescents’ evaluations of different forms of bystander support as this can provide insights into their own likelihood to provide that type of support to a target of cyberbullying.

In studies that have examined evaluations of bystander behaviours in cyberbullying, adolescents are generally supportive of bystanders who defend targets of cyberbullying [15], but they also support bystanders who remain passive [16,17,18]. However, little is known about how helpful they perceive positive and negative interventions from bystanders in the form of constructive and aggressive behaviours for targets of cyberbullying. Understanding their perceptions of bystander support is important to tailor prevention activities aimed at encouraging helpful responses in bystanders of cyberbullying in order for interventions to be meaningful and perceived as helpful by adolescents.

### 1.2. Sources of Support in Cyberbullying

While evaluations of bystander behaviours can help predict how a bystander might respond to cyberbullying, there are other contextual factors to consider. Specifically, a bystander’s relationship with the parties involved in cyberbullying can complicate their likelihood to respond to cyberbullying and their chosen response (e.g., to do nothing, support the target, or encourage the cyberbully). For instance, when the target is an acquaintance of the bystander, but the cyberbully is a friend, bystanders are more likely to encourage the cyberbully [19]. Other research has shown that adolescents are more likely to defend a target when they feel supported by their friends [20]. In other situations, adolescents are more likely to stay passive when they are friends with a perpetrator of cyberbullying [21] or an acquaintance of the individuals involved [20] and not get involved in the situation. However, when confronted with hypothetical situations, adolescents report that if they were friends with the perpetrator of cyberbullying, they would confront their friend and ask them to stop [22]. Conversely, if they were acquaintances with a cyberbully, they would ignore the situation and take on a passive role [22]. In the same study, when adolescents read hypothetical cyberbullying scenarios where the bystander and the target of cyberbullying were friends, constructive support such as reporting the incident, asking the cyberbully to stop, or providing comfort were all considered likely responses [22]. This research highlights the influence of a bystander’s relationship with both the cyberbullies and targets involved in cyberbullying on the responses they choose to have when faced with a cyberbullying event (i.e., to remain passive, to confront the cyberbully, to provide comfort, etc.). However, little is known about how a bystander’s relationship with the target can influence adolescents’ evaluations of different forms of constructive (e.g., politely asking the cyberbullying to stop or providing comfort) and aggressive (e.g., exposing the cyberbully or biting back at them) support.

In the realm of relationships, the public nature of social media can also complicate matters. Specifically, as adolescents’ social interactions extend to the online world, adolescents will likely interact with individuals outside of their peer group (e.g., social media influencers; [23,24]. However, little is known about how public figure status impacts perceptions of bystander support in adolescents. In studies that examined celebrity feuds, it was shown that celebrities even respond to fan-based cyberbullying in negative (e.g., “biting back”) and positive ways (e.g., promoting a “don’t bully, be happy” message; [5]. While adolescents show feelings of uneasiness in response to celebrity engagement in cyberbullying [9], they convey approval of public bashing of celebrities [25]. Given the influential role of public figures, these responses can shape the attitudes and behaviours of impressionable adolescents (e.g., [26,27]), who, in turn, may model these behavioural responses when they are witnessing cyberbullying. Accordingly, it is important to understand how adolescents perceive this bystander support from public figures to know how likely they are to model their behaviours. It can also provide some indications of how adolescents perceive those forms of constructive and aggressive support, should they be receiving it if they were targets of cyberbullying.

### 1.3. The Present Study

While some research has highlighted how adolescents evaluate active and passive support from friends and acquaintances, little is known about how they evaluate specific forms of constructive and aggressive support from public figures in addition to friends and acquaintances. Adolescent perceptions of these different approaches to bystander support are important to examine because, while both are enacted with the intention to help a target of cyberbullying, it is unclear if they are perceived as helpful or hurtful by targets of cyberbullying and social media users. As a result, the present study examines how adolescents evaluate both constructive and aggressive types of bystander behaviours according to the bystander’s relationship with a target of cyberbullying. Because adolescents generally provide positive evaluations of bystanders who defend targets of cyberbullying by reporting a post or asking a cyberbully to stop (e.g., [15]), we hypothesized that adolescents would generally evaluate constructive types of bystander support as helpful, regardless of the bystander’s relationship with the target. Moreover, previous research showed that bystanders are likely to side with the cyberbully if they were friends with them (e.g., [22]). As a result, they may approve of more aggressive behaviours. In the same vein, we hypothesized that adolescents would be likely to perceive aggressive forms of support as helpful from friends of the target, but they would find it harmful when directed by acquaintances of the target. Finally, because adolescents are likely to approve of instances of celebrity bashing [25], we hypothesized that they would perceive aggressive types of support from public figures as helpful.

## 2. Methods

### 2.1. Participants

Ninety-nine adolescents between 13 and 17 years old (*M*_age_ = 14.42; *SD* = 1.35; 63.3% female) participated in this study. Participants were recruited from a university research database of families with adolescents in the Montreal area of Quebec, Canada that had previously expressed interest in participating in research, and from advertisements across Canada and parts of the United States. Parents completed a short socio–demographic questionnaire. Parents described their families primarily as Canadian (54.2%), followed by European (22.5%), and the remaining participants (23.3%) described themselves as of Latin American, Asian, Middle Eastern or African descent. The majority of participants were from middle-class families. Recruitment was deemed complete when the appropriate number of participants was reached for statistical power according to an analysis using G*Power (Version 3.1, Mannheim University, Germany).

### 2.2. Materials

#### 2.2.1. Video–Vignettes

**Figure 1:**
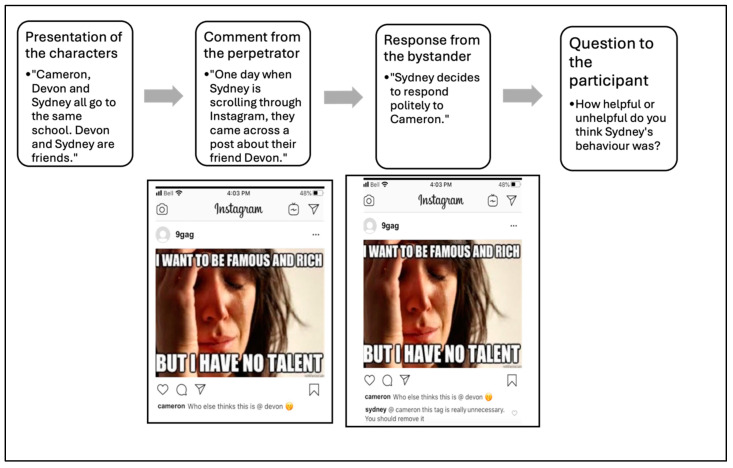
Example of video content. Each video followed the same sequence of events and provided voiceovers describing interactions and events.

During data collection, adolescents viewed a total of 9 videos, each depicting a hypothetical public cyberbullying situation on Instagram and a different form of constructive or aggressive bystander support. The videos depicted a fictional event and were created by the researchers for the purposes of the study. The videos involved screenshots with voiceovers of the sequence of events. An example is presented in Figure 1. None of the videos specified the number of comments, shares, or likes on the perpetrators’ posts or the bystanders’ responses to control for social influences on participant responses. In all videos, names were kept gender neutral (e.g., Cameron, Riley, Jamie, Ali, etc.). Age was not mentioned to ensure that all participants in the sample’s age range could relate to the events depicted in the videos. Each video was approximately one minute in duration and illustrated an adolescent being cyberbullied by a perpetrator in an Instagram post or story, and a bystander supporting the adolescent being cyberbullied (i.e., by commenting on their response on the perpetrator’s post or reposting the story with their response). Participants were randomly assigned to either the constructive (*n* = 52) or aggressive (*n* = 47) support condition. Targeted behaviours for the constructive and aggressive forms of support were developed based on Ouvrein et al.’s (2019) [5] cyberbullying experience and behavioural coping frames. Constructive forms of bystander support involved a demonstration of empathy towards the target, politely asking the cyberbully to stop, or attempting to cheer up the target by providing a positive view of the situation. Aggressive forms of bystander support involved publicly calling out or exposing the cyberbully for their actions, biting back at the cyberbully, or placing blame on one of the shortcomings of the cyberbully. Moreover, the bystander was either an acquaintance (3 videos), a friend (3 videos), or a made-up public figure (3 videos) in relation to the target of cyberbullying. Gender-neutral names were used in all videos.

Evaluations of Bystander Support. After viewing each video in their assigned condition (constructive or aggressive support), the researchers asked adolescents about their evaluation of each bystander’s behaviour (“How harmful or helpful do you think [bystander’s] behaviour is?”) on a Likert-type scale from −2 (very hurtful) to 2 (very helpful). These evaluations measured adolescents’ perceptions of bystander support from the cyberbullying target’s perspective.

#### 2.2.2. Procedure

This study was approved by McGill University’s Research Ethics Board (Tier III). The study was conducted online through Zoom. After parents provided informed consent though email, a short socio–demographic questionnaire was sent to them via Qualtrics and video calls were scheduled with the adolescents to complete the study. Prior to beginning the study, participants were randomly assigned to the constructive or aggressive support condition. A research assistant ran each video call with participants. The study began with the research assistant asking adolescents for their assent. Next, the research assistant shared their screen with the participants to show them the videos. After showing each of the nine videos in their assigned condition, the research assistant asked adolescents how they evaluated the behaviour of the bystander in the respective video. The order of the videos was counterbalanced across subjects. At the end of the study, adolescents were debriefed and provided with resources for dealing with cyberbullying.

## 3. Results

### 3.1. Data Analysis and Assumption Checks

The objective of the data analysis was to examine adolescents’ evaluations of bystander support during cyberbullying according to the form provided and their relationship with the target. Data were analyzed with IBM SPSS Statistics (Version 29.0.1.1 for Macintosh, Armonk, NY, USA). A two–by–three–by–three mixed analysis of variance (ANOVA) was run with the bystander’s form of support (constructive or aggressive) as a between-subjects variable, their relationship to the target (acquaintance, friend, or public figure) and the bystander’s targeted behaviour (e.g., to bite back, to show empathy, etc.) as within-subject variables, and their rating of harmfulness or helpfulness (measured continuously) of the bystander’s behaviour as the outcome variable, and age as a covariate. All underlying assumptions were met except for Mauchly’s test of sphericity, which was violated in some instances. When the assumption was violated, the more conservative Greenhouse–Geisser is reported as a test result.

### 3.2. Adolescents’ Evaluations of Bystander Support

Table 1 shows the means and standard deviation for all evaluations according to the bystander’s relationship with the target and type of support. There was no significant main effect of the bystander’s targeted behaviour, *F*(2, 186) = 0.23, *p* = 0.79, partial eta^2^ < 0.01. There was, however, a significant main effect of the relationship of the bystander to the target of cyberbullying, *F*(2, 186) = 8.98, *p* < 0.01, partial eta^2^ = 0.09. Post hoc tests showed that support from friends (*p* = 0.01), and public figures (*p* < 0.01), were evaluated as significantly more helpful than support from acquaintances. There was no significant difference between support from friends and public figures when controlling for the bystander’s targeted form of support.

There was a significant three-way interaction between the type of support provided, the bystander’s relationship with the target of cyberbullying, and the bystander’s targeted behaviour (i.e., the specific form of constructive or aggressive support), *F*(4, 372) = 21.31, *p* < 0.01, partial eta^2^ = 0.19. To locate the significant differences in evaluations, post-hoc simple two-way interactions were conducted for each group on the between-subjects variable (type of bystander support). For both constructive bystander support, *F*(3.25, 159.17) = 5.61, *p* < 0.01, partial eta^2^ = 0.10, and aggressive bystander support, *F*(2.81, 123.66) = 18.80, *p* < 0.01, partial eta^2^ = 0.30, there was a significant interaction between the bystander’s specific behaviour and their relationship with the target of cyberbullying. To better understand the significant differences, post-hoc tests of simple main effects were run on each of the types of bystander support and for each relationship type (acquaintance, friend, and public figure).

**Figure 2:**
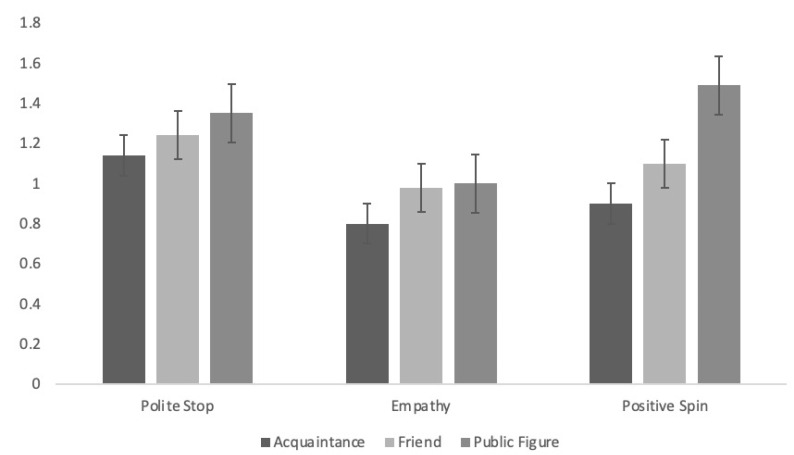
Evaluations of constructive bystander support according to the bystander’s behaviour and their relationship to the target of cyberbullying. Note: Scores can range from −2 (very harmful) to 2 (very helpful).

For constructive support (Figure 2), bystanders who were acquaintances were evaluated significantly differently depending on their specific behaviour, *F*(2, 100) = 4.36, *p* = 0.02, partial eta^2^ = 0.08. Politely asking the cyberbully to stop was evaluated as significantly more helpful than a show of empathy towards the target (*p* = 0.4) but was not significantly different from bystanders who tried to give the situation a positive spin in favour of the target (*p* = 0.10). There were no significant differences between bystanders who showed empathy or tried to give the situation a positive spin (*p =* 1.00). For bystanders who were friends with the target, there were no significant differences in the perceived helpfulness of their supportive behaviour, *F*(2, 98) = 2.13, *p* = 0.12, partial eta^2^ = 0.04. However, there were differences in how constructive support was evaluated from bystanders who were public figures, *F*(1.77, 88.29) = 9.74, *p <* 0.01, partial eta^2^ = 0.16. Public figures who tried to give the situation a positive spin in favour of the target (*p <* 0.01) and those who politely asked the cyberbully to stop (*p* = 0.03) were evaluated as more helpful than those who showed empathy, but there were no significant differences in helpfulness ratings between giving the situation a positive spin or politely asking the cyberbully to stop (*p* = 0.59).

**Figure 3:**
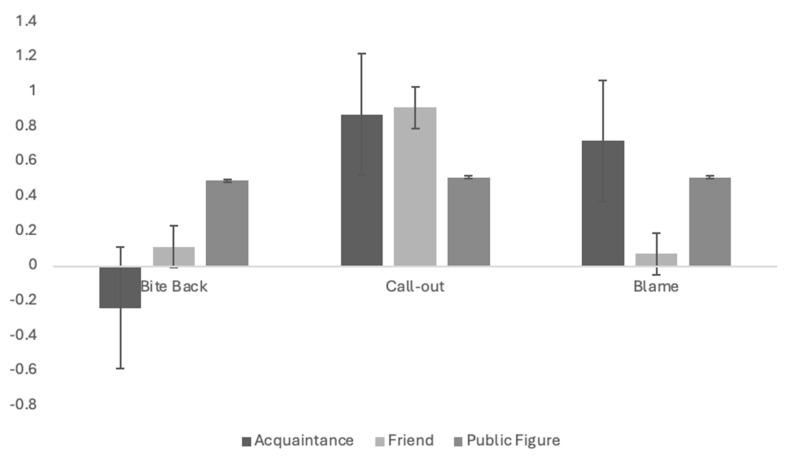
Evaluations of aggressive bystander support according to the bystander’s behaviour and their relationship to the target of cyberbullying. Note. Scores can range from −2 (very harmful) to 2 (very helpful).

For negative bystander support (Figure 3), there were significant differences in how the behaviours of acquaintances were evaluated, *F*(2, 90) = 29.85, *p <* 0.01, partial eta^2^ = 0.40. Biting back at the cyberbully was evaluated as more harmful than both calling out the cyberbully (*p <* 0.01) and blaming the cyberbully (*p <* 0.01). There were no significant differences between evaluations of calling out the cyberbully or blaming them (*p* = 0.91). Both were evaluated as helpful rather than harmful. There were also significant differences in how the negative support of friends was evaluated, *F*(1.68, 73.94) = 13.35, *p <* 0.01, partial eta^2^ = 0.23. Negative bystander support from friends was generally viewed as positive with calling out the cyberbully as more helpful than blaming the cyberbully (*p <* 0.01) or biting back at them (*p <* 0.01). There were no significant differences between friends of the target that blamed the cyberbully or bit back at them (*p* = 1.00). Finally, there were no significant differences in how negative support from public figures was evaluated, *F*(2, 92) = 0.01, *p =* 0.99, partial eta^2^ = 0.01.

## 4. Discussion

The current study examined adolescents’ evaluations of the helpfulness and harmfulness of different bystander responses to cyberbullying. Significant differences were found in how adolescents evaluated the behaviours of bystanders who engaged constructively and aggressively. Generally, as hypothesized, adolescents evaluated constructive forms of support as helpful regardless of the bystander’s relationship with the target. However, evaluations of the helpfulness of specific constructive and aggressive bystander behaviours were significantly different according to the bystander’s relationship with the target of cyberbullying.

When the bystander was acquainted with the target, perceptions of helpfulness varied for both constructive and aggressive supportive behaviours. For constructive forms of support, adolescents perceived bystanders who politely asked the cyberbully to stop as more helpful than those who demonstrated an empathetic response to the target. Putting a positive spin on the situation was not viewed as significantly more helpful or harmful than showing empathy or politely asking the cyberbully to stop. These findings show that emotional forms of support (e.g., showing empathy) are supported when provided by friends but not acquaintances. In existing studies on cyberbullying interventions, the promotion and teaching of empathy was shown to be an effective way to have bystanders defend targets of cyberbullying [28]. However, while promoting empathy can encourage bystanders to engage in supportive behaviours, our results suggest that showing empathic responses may not be the most helpful avenue depending on their relationship with a target of cyberbullying. For aggressive responses to cyberbullying, adolescents perceived calling out or exposing the cyberbully as helpful, but biting back or placing blame on them as equally harmful. This supports our hypothesis that more direct aggressive responses from acquaintances are not generally perceived as helpful [4,22]. Our findings also show that adolescents are likely to find it helpful to defend a target of cyberbullying by interacting respectfully with the cyberbully, even if they are not close friends with them.

When the bystander was a friend to the target, differences in perceptions of helpfulness were only significant for aggressive forms of support, as all forms of constructive support were seen as helpful. Adolescents perceived bystanders who called out or exposed the cyberbully as more helpful than those who blamed the cyberbully or bit back at them. There were no significant differences between perceptions of helpfulness for bystanders who placed blame on the cyberbully or bit back at them. As opposed to aggressive support from acquaintances that was seen as harmful, direct aggressive responses from friends, while not significantly different from each other, were generally evaluated as helpful. Accordingly, just as adolescents are likely to support bystanders who attack a target when they are friends with the cyberbully [22], they are also likely to support bystanders who act aggressively towards cyberbullies when they are friends with the target. According to Latané & Darley’s (1970) Bystander Intervention Model [11], these findings have problematic implications for the escalation of cyberbullying if bystanders’ perceptions of these behaviours are associated with their actual interventions when witnessing cyberbullying.

Finally, when the bystander was a public figure, differences in perceptions of the helpfulness of specific responses to cyberbullying were only significant for constructive forms of support. Adolescents perceived public figures who put a positive spin on the situation for the target and those who politely asked the cyberbully to stop as more helpful than showing empathy. Moreover, negative forms of support were generally evaluated as helpful, and no significant differences were found between evaluations of specific negative behaviours. Currently, little is known about how adolescents perceive bystander support from public figures. Some existing findings show that adolescents report feelings of unease when witnessing negative behaviours from celebrities [9] and this may explain why some forms constructive support were evaluated positively. However, adolescents also report support for public aggressive interactions between celebrities [25]. Accordingly, further research is needed to better understand the dynamic between adolescents’ positive evaluations of aggressive support and their negative feelings toward celebrity engagement in cyberbullying. From a practical standpoint, in addition to implications for the escalation of cyberbullying, these findings have implications for the well-being of targets and adolescents who witness cyberbullying, specifically in relation to the negative feelings adolescents report in response to aggressive responses from public figures online [9]. These findings highlight the importance of engaging the online community, including public figures, to use their online presence to promote respectful and helpful behaviours on social media.

Theoretically, our findings add to the growing body of work that contextualizes Latané & Darley’s (1970) Bystander Intervention Model [11] to online-mediated contexts. Specifically, our findings suggest that relationships with a bystander do matter when deciding if and how to intervene as a bystander. For instance, adolescents find it helpful for bystanders they do not know personally (i.e., acquaintances and public figures) to politely ask a cyberbully to stop, and for a friend to provide emotional support. As a result, bystanders’ method of intervention may vary according to their relationship with the target of cyberbullying.

The results from our study have similar practical implications. Many interventions promote defending behaviours from bystanders in cyberbullying (e.g., [10]). However, no study to our knowledge has assessed how helpful adolescents find these types of interventions from bystanders. Our findings show that adolescents do not evaluate all forms of support as helpful from bystanders and that bystanders should consider their relationship with a target of cyberbullying before deciding how best to support them.

### Limitations and Future Directions

While the current study presents novel findings on adolescents’ evaluations of constructive and aggressive behaviours to support targets of cyberbullying, there are some limitations. First, hypothetical situations rather than real-life experiences were used. Future research should compare perceptions of the helpfulness of bystander support in both hypothetical and adolescents’ reported real-life situations to validate the relationship between perceptions and actions highlighted by the Bystander Intervention Model. In addition, some individual factors such as adolescents’ personal experiences as perpetrators, targets, or bystanders of cyberbullying, gender identity, and age may influence their evaluations and could be considered in future research. Moreover, adolescents were asked how they evaluated the bystander’s type of support on a continuous scale, but they were not asked why they provided those evaluations. Future research should contextualize adolescents’ perceptions of bystander support to provide insights into the underlying mechanisms guiding their evaluations (e.g., moral disengagement mechanisms; [17]).

The videos manipulated the target’s relationship with the bystander as either being a friend, an acquaintance, or a public figure. However, they did not consider whether the bystander was a complete stranger to the target. Previous research found that adolescents believed that strangers to a target or perpetrator of cyberbullying were likely to ignore cyberbullying [22]. Nevertheless, given the public nature of social media, it is likely that strangers regularly witness cyberbullying. Accordingly, future research should consider extending evaluations of helpfulness of bystander support to strangers. In addition, the videos emphasized that the public figures in question were public figures, but they were not known individuals. Future research could have adolescents evaluate actual known events where public figures engaged in cyberbullying as bystanders or examine associated variables such as levels of celebrity worship [26,27] to understand how adolescents would be influenced by public figures. Finally, all videos depicted public instances of cyberbullying on Instagram, but future research could extend to other social media platforms that adolescents are likely to engage with, such as WhatsApp, Snapchat or TikTok. Their activity levels and/or proficiency on each platform could also be measured to examine if they influence perceptions.

## 5. Conclusions

Findings from this study provided unique insights into how friends, acquaintances and public figures can support adolescent social media users who are targets of cyberbullying by enhancing their well-being and their sense of safety on Instagram. Adolescents generally supported constructive types of behaviours that are more instrumental (i.e., asking a cyberbully to stop) from acquaintances and public figures as opposed to emotional (i.e., showing empathy). However, some aggressive types of support, such as exposing a cyberbully or biting back at them, were evaluated as helpful when engaged in by friends. To avoid escalating cyberbullying events and reduce negative feelings for targets of cyberbullying, prevention activities can focus on the promotion of constructive responses to cyberbullying that are either instrumental (from acquaintances and public figures) or instrumental and emotional (from friends) and are perceived as helpful from adolescents. Overall, our findings have important implications for promoting safe and meaningful interventions for adolescent bystanders who witness cyberbullying according to their relationship with a target.

## Figures and Tables

**Table 1:** Descriptive statistics (Means (Standard Deviation)) for evaluations of the helpfulness of bystander support according to the bystander’s relationship with the target and the type of support on Likert scale.

	Acquaintance	Friend	Public Figure
Constructive			
Polite stop	1.14 (0.64)	1.24 (0.63)	1.34 (0.60)
Empathy	0.80 (0.88)	0.98 (0.96)	1.00 (0.78)
Positive spin	0.90 (0.61)	1.10 (0.84)	1.50 (0.54)
Aggressive			
Bite back	−0.27 (0.86)	0.11 (0.98)	0.49 (1.10)
Call-out	0.87 (1.10)	0.91 (1.20)	0.56 (1.06)
Blame	0.76 (0.93)	0.07 (1.07)	0.49 (1.04)

Note. Scores can range from −2 (very harmful) to 2 (very helpful).

## Data Availability

The raw data supporting the conclusions of this article will be made available by the authors on request.
